# AntimiR uptake by human proximal tubule epithelial cells is predominantly by macropinocytosis

**DOI:** 10.1038/s41598-025-16522-3

**Published:** 2025-08-29

**Authors:** Emily K. Glover, Rolando Berlinguer-Palmini, Emily R. Thompson, Colin Wilson, Laura Denby, Gary Reynolds, Simi Ali, Martin Lowe, Rachel Lennon, Neil S. Sheerin

**Affiliations:** 1 Translational and Clinical Research Institute, Faculty of Medical Sciences, Newcastle University, Newcastle Upon Tyne, UK; 2 Bioimaging Unit, Faculty of Medical Sciences, Newcastle University, Newcastle Upon Tyne, UK; 3 Centre for Cardiovascular Sciences, University of Edinburgh, Edinburgh, UK; 4 Biosciences Institute, Newcastle University, Newcastle Upon Tyne, UK; 5 Centre for Immunology and Inflammatory Diseases, Mass General Research Institute, Harvard Medical School Boston, Boston, USA; 6 Faculty of Biology, Medicine and Health, School of Biological Sciences, University of Manchester, Manchester, UK; 7 Division of Cell Matrix Biology and Regenerative Medicine, University of Manchester, Manchester, UK

**Keywords:** Endocytosis, Macropinocytosis, AntimiR, Proximal tubule epithelial cells, Trafficking, antimiR, Membrane trafficking, Nephrology, Drug delivery

## Abstract

The ease with which microRNA inhibitors (antimiRs) can be delivered varies with the intended target cell type and tissue. AntimiRs are of interest as potential therapeutics for kidney conditions, including ischaemia–reperfusion injury in transplantation. During ex-situ human kidney perfusion, antimiRs are delivered to the proximal tubule epithelium without the use of transfection reagents by an endocytic process. Here we investigate whether antimiR uptake by proximal tubule epithelial cells (PTEC) occurs *in vitro*, without transfection reagents, and which endocytic entry mechanism is responsible. PTEC were isolated from human kidneys declined for transplantation and maintained in culture. PTEC were shown to take up antimiR through detection of increased fluorescent signal associated with the antimiR label. A vesicular pattern of uptake was demonstrated on fluorescence microscopy, in keeping with endocytic uptake. Endocytosis was confirmed by co-occurrence of the antimiR with endocytic markers and temperature dependence of uptake. Megalin inhibition with receptor associated protein to target receptor-mediated endocytosis had no effect on antimiR uptake, whereas the macropinocytosis inhibitor 5-(*N*-ethyl-*N*-isopropyl)-amiloride reduced antimiR uptake. Our results demonstrate antimiR can be delivered to primary human PTEC *in vitro* without the use of transfection reagents and support macropinocytosis as the dominant pathway of antimiR entry.

**Supplementary Information:**

The online version contains supplementary material available at 10.1038/s41598-025-16522-3.

## Introduction

MicroRNAs (miRNAs) are short non-coding regulatory RNA sequences that target specific messenger RNA (mRNA) sequences, typically at the 3′-untranslated region through complementary binding. Through this binding, miRNAs downregulate their target mRNAs by preventing initiation of translation or by triggering target mRNA degradation^1,2^. A single miRNA is complementary to multiple mRNA sequences and in this way they are key regulators of cellular processes. Modulating miRNA action through use of mimics or inhibitors (antimiRs) is therefore attractive as a potential therapeutic intervention and antimiRs have reached clinical trials including to target kidney disease (NCT02855268)^2,3^. Chemical modifications have improved resistance of antimiRs to nucleases and the affinity of binding to target sequences^1,2,4^. Additionally, the phosphorothiate backbone of the antimiR is thought to enable naked uptake, without the use of transfection reagents, referred to as “gymnosis”^5–7^.

One of the challenges in using antimiR therapies is ensuring effective delivery to the intended target cells^2,8^. The kidney, along with the liver, is one of the easiest organs to target with systemic administration of oligonucleotides^9^. There are several studies in mice in which naked antimiR has been delivered to the kidney by systemic administration, without the need for a particular carrier molecule^10,11^. As oligonucleotides are smaller than 6 nm, they are freely filtered from the blood at the glomerulus and pass through to the tubular lumen^8,10^. The proximal tubule epithelium is responsible for reabsorbing much of the glomerular filtrate so unsurprisingly is the main site of oligonucleotide uptake within the kidney^11–15^. The proximal tubule has been shown to be the main site of antimiR uptake during ex-situ human kidney perfusion, with uptake occurring to a lesser extent in the cells of the glomerulus and blood vessel walls^16^.

AntimiR uptake into the proximal tubule epithelium is an active endocytic process as it is does not occur at 4 °C and fluorescently labelled antimiR colocalises with markers of the early and late endosome^16^. The importance of active transport is consistent with data from HeLa cells where both cooling and energy depletion prevented nucleic acid uptake^17^. However, endocytic transport encompasses a variety of mechanisms for cellular uptake and the mechanism of oligonucleotide uptake by proximal tubule epithelial cells (PTEC) is unknown and a significant gap in our understanding^9,18,19^.

There are two major mechanisms of uptake into PTECs, one is uptake via megalin, a large transmembrane receptor internalized by clathrin-mediated endocytosis^20,21^. The other is macropinocytosis, which in contrast, is a more ubiquitous process in which much larger vesicles are generated by cell protrusions indiscriminately encapsulating large volumes of extracellular fluid^18,19,22–24^.

Using primary human PTEC *in vitro* we investigate the involvement of these two endocytic pathways in the uptake of naked antimiR. We identify macropinocytosis as the dominant pathway for proximal tubule uptake of antimiR.

## Materials and methods

### Cell isolation and culture

Human kidneys declined for transplantation and with appropriate consent for research were used for isolation of primary PTECs. All methods were performed in accordance with the relevant guidelines and regulations. Cold ischaemic time for kidneys used was limited to 30 h and only kidneys from donors under 70 years old were used. The PTEC isolation method was adapted from Brown et al.^25^ and Bajaj et al.^26^ (Supplementary Methods, Supplementary Fig. 1, Supplementary Fig. 2).

**Fig. 1:**
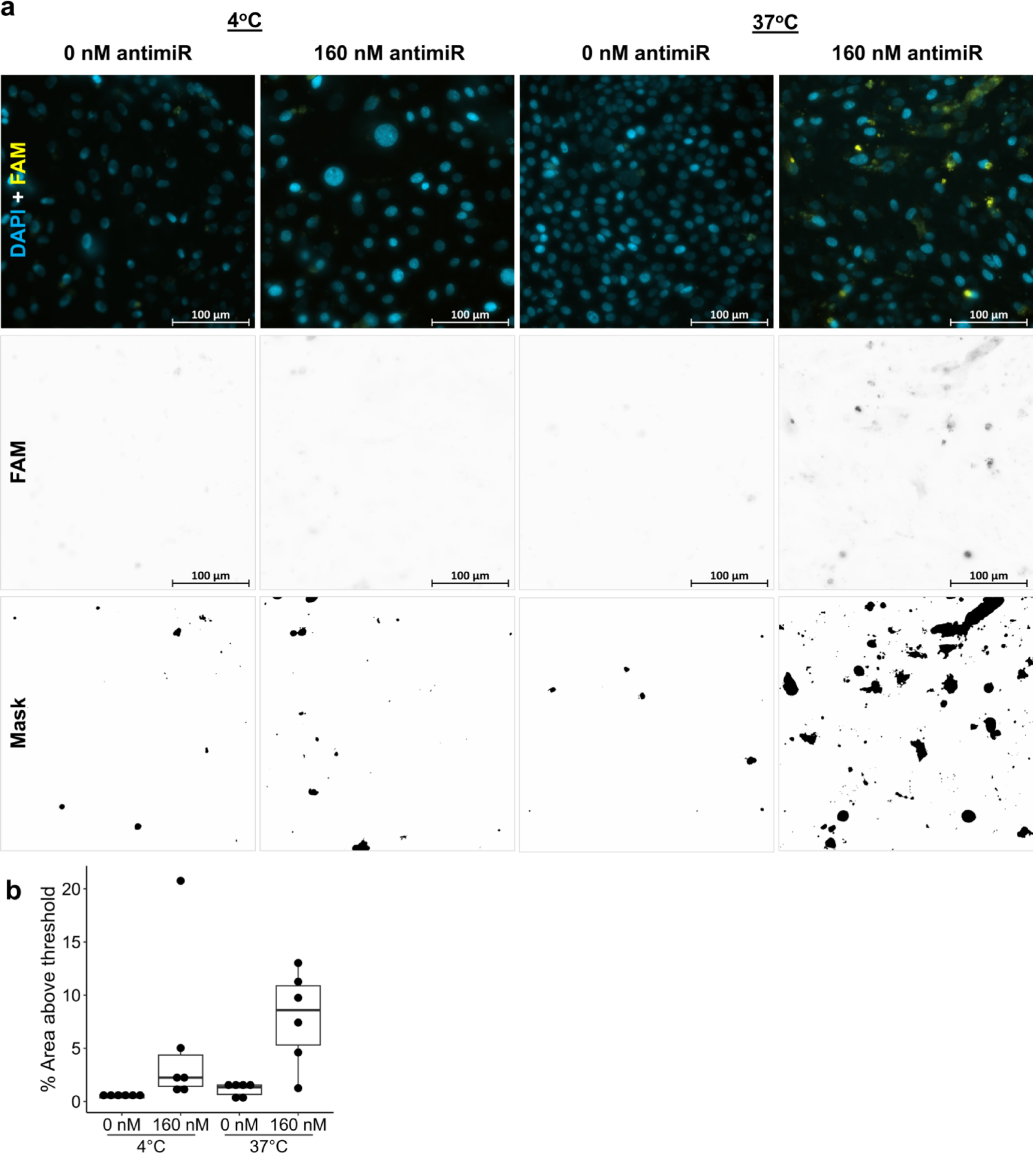
Fluorescence microscopy of naked FAM-labelled antimiR (yellow) uptake into human primary proximal tubule epithelial cells (PTEC) after 8 h of exposure to antimiR at 4 °C or 37 °C. PTEC were maintained on transwell inserts and 160 nM antimiR was added to apical compartment. Nuclei are counterstained with DAPI (cyan) and control cells, not exposed to antimiR, are shown for each temperature. Images shown in (**a**) are representative of the 6 images acquired at random for each condition using ZEISS AxioImager with X40 lens. FAM signal images are inverted for clarity beneath and the associated binary masks of areas deemed “positive” given below. Quantification of individual images are presented as points on the boxplots in (**b**). All data is from one biological repeat.

Freshly isolated or thawed cryopreserved PTEC were seeded onto the apical side of transwell inserts (Sarstedt, 0.4 µm pore size, transparent PET membranes with pore density 2 × 10^6^ pores/cm^2^) at a density of 226 666 viable cells/cm^2^ and maintained in culture without passage for up to 10 days. Cells were seeded directly onto membranes without the use of extracellular matrix coatings. Maintenance media (DMEM/F-12 50/50 (Sigma Aldrich D8437, Corning 10-092-CV) with 15 mM HEPES and sodium bicarbonate and supplemented to 4.0 mM L-glutamine and 1% (v/v) Penicillin/Streptomycin and with REGM SingleQuot Kit Supplement with growth factors (CC-4127 Lonza)) was changed in both apical and basolateral compartments 24 h after seeding, then every 48–72 h subsequently. PTEC were used for experiments once confluent, usually from 4 days after seeding. Cells were maintained at 37 °C with 5% CO_2_ in a humidified incubator unless otherwise stated.

### Assessment of endocytosis

Fluorescein amidites (FAM) labelled scrambled (no target sequence, molecular weight 5553.5 g/mol) antimiR (Qiagen YC10202062-FZB) was diluted to working concentrations (10–160 nM) with maintenance media and added to the apical compartment. No transfection reagents were used to deliver the antimiR. Texas Red-labelled 10 kDa dextran (ThermoFisher D1828) was applied to the apical compartment at a concentration of 1 mg/mL. Human albumin was conjugated with either fluorescein isothiocyanate (FITC; Sigma A9771) or Texas Red (ThermoFisher A23017) and applied to the apical compartment at a final concentration of 0.1 mg/mL. To assess the effect of temperature on antimiR uptake media was cooled to 4 °C before application and cells maintained at this temperature for the duration of treatment.

### Pathway inhibition

PTEC viability in response to concentrations of 5-(*N*-ethyl-*N*-isopropyl)amiloride (EIPA; Santa Cruz Biotechnology sc-202458) was assessed by flow cytometry, light microscopy and nuclear appearance on fluorescence microscopy (Supplementary Methods). Receptor-associated protein (RAP) with a polyhistidine tag was made in house in the Lowe lab^27^. RAP and EIPA were diluted to working concentrations with maintenance media. EIPA was added to both apical and basolateral compartments whereas RAP was only added to apical compartment. Pretreatment was for 1 h before addition of antimiR, albumin or dextran to the apical compartment with continued inhibitor exposure.

### Immunofluorescence

PTEC maintained on 24-well transwell inserts were washed with phosphate-buffered saline (PBS) and fixed with 4% methanol-free paraformaldehyde (PFA) in PBS for 10 min. Fixed cells were washed, permeabilized by incubation with 0.1% Triton-X in PBS for 4 min, washed and blocked with 10% goat serum in PBS for 1 h at room temperature. AntimiR treated cells were incubated with rabbit anti-RAB7 antibody (Abcam ab137029) at a concentration of 55 ng/mL overnight at 4 °C. Matched controls not exposed to antimiR were incubated with isotype control (R&D Systems rAB-105-C) at an equivalent concentration. Cells were then washed and incubated for 1 h with AF594-conjugated anti-rabbit secondary (Biolegend 406,418) at a concentration of 5 mg/mL. After washing, membranes were cut from inserts and mounted with VectaShield Antifade Mounting media with DAPI (2B Scientific H-1200-10). The cover slip was sealed. If no antibodies were used, membranes proceeded directly to mounting after fixation.

### Widefield fluorescence microscopy image acquisition and signal quantification

AntimiR, albumin and dextran uptake was quantified by widefield fluorescence microscopy using a standardised approach to image acquisition. Images were acquired at random of confluent areas of cells guided by nuclear DAPI signal and blinded to fluorescent signal in the other channels. Images were acquired with ZEISS AxioImager with X40 lens.

Single channel images were converted to .tif using ZEISS Zen Microscopy Software (RRID: SCR_013672) with the same display settings applied to each repeat within a batch. In Fiji (RRID:SCR_002285), a threshold level was set using the control autofluorescence images to have at least 95% of pixels in the control image below the threshold. The area of images above this threshold was quantified from a binary mask and when an uptake inhibitor (EIPA or RAP) was used, these values are expressed relative to cells not exposed to the inhibitor.

### Confocal microscopy image acquisition and colocalisation

Images for co-occurrence analysis between antimiR, albumin, dextran and RAB7 were acquired by Leica SP8-gSTED 3X microscope using X63/1.4NA oil lens. A z-stack was acquired using optimal voxel size (X,Y ≤ 44 nm, Z = 130 nm) as calculated using Nyquist calculator. Z-stacks of albumin and dextran uptake consisted of 20 slices and those for RAB7 immunofluorescence were 13 slices. Areas demonstrating marker uptake were targeted for image capture in the treated cells. Confocal microscopy image acquisition was standardized and within each biological repeat images were captured with identical setting for control and treated cells to allow comparison.

Deconvolution and colocalisation analysis were performed in Huygens Software (www.SVI.nl-RRID:SCR_014237). Firstly, images were deconvolved with identical settings used for all images within each biological repeat. Deconvolution settings were optimised against untreated cells. Pixels to include in colocalisation analysis of images of treated cells were all above the threshold values set by the highest control image (excluding outliers). Images with excessive artefact or minimal signal above control thresholds were excluded to leave a minimum of 5 stacks included per biological repeat. Colocalisation was assessed by Manders’ overlap coefficient.

### *LRP2* knockdown

PTEC were transfected with siRNA targeting *LRP2* (megalin; SCBT sc-40103) or control siRNA (SCBT sc-37007) using Lipofectamine RNAimax transfection reagent (ThermoFisher 13778). Transfection mix was produced by combining equal volumes of OPTI-MEM® mixed with Lipofectamine in a 50:3 ratio and siRNA stock diluted with OPTI-MEM®. PTEC for transfection were seeded onto 12-well inserts and transfected when confluent by addition of 30 µL of transfection mix to 170 µL of maintenance media in the apical compartment. Apical compartment media was topped up to 300 µL after 4 h to give a final siRNA concentration of 200 nM. Media in both compartments was replaced 24 h after transfection. Fluorescently labelled albumin (0.1 mg/mL) or antimiR (40 nM) were added to apical compartment 96 h after transfection for 24 h of treatment.

### Flow cytometry

Cells were detached with Accutase (Millipore SCR005), washed and resuspended in flow cytometry buffer (2% fetal bovine serum in PBS). The amine-reactive fluorescent dye (Zombie Violet, Biolegend 423113) was used to differentiate live and dead cells. Cells were fixed with 4% PFA in PBS for 15 min before washing twice with PBS.

To assess total (surface and intracellular) megalin expression, harvested cells were incubated with Fc Receptor block before amine-reactive fluorescent dye. After fixation, cells were permeabilised by incubation with 0.2% Trixton-X in PBS for 15 min on ice, then washed and incubated with AF647-conjugated anti-LRP2 (R&D Systems FAB9578) or isotype control (R&D Systems IC002R) at 2 µL in 98 µL flow cytometry buffer for 30 min on ice. Finally, cells were washed and resuspended in flow cytometry buffer.

Analysis was by the BD Symphony A5 with downstream analysis in FlowJo™ Software (RRID:SCR_008520).

### Statistics

Repeated measures analysis of variance (ANOVA) was used to compare means of > 2 groups. Paired t-test was used to compare means of 2 groups with Benjamini–Hochberg correction for multiple comparison when appropriate. *P* < 0.05 was taken as statistically significant. Statistical analysis was performed with R Core Team 2023 (R Foundation for Statistical Computing, Vienna, Austria (https://www.R-project.org/)).

## Results

### AntimiR uptake by PTEC is temperature dependent

To confirm if the mechanism for antimiR uptake into PTEC is an active process, the effect of temperature was explored by treating cells with FAM-labelled antimiR in warm and cold conditions (Fig. 1). In PTECs incubated for 8 h with 160 nM antimiR at 4 °C, a median (interquartile range) of 2.24% (2.94%) of pixels were above the threshold set as positive for FAM signal (Fig. 1b). For PTECs treated with antimiR at 37 °C, more uptake was indicated by an increase in FAM positive area to 8.58% (5.57%, Fig. 1b). In cells not incubated with antimiR, FAM signal was lower, as expected, at 1.34% (0.89%) at 37 °C and 0.52% (0.29%) at 4 °C (Fig. 1b), which reflects a degree of autofluorescence. Therefore, confirming uptake was temperature dependent, in keeping with an active process. The approach of quantifying FAM signal to binary masks of positive areas is demonstrated for examples images in Fig. 1a. Although cell viability was reduced after 8 h at 4 °C (79.5%) compared to 37 °C (91.5%), this is a modest reduction and would not fully explain the reduction in antimiR uptake observed in cold conditions. Additionally, the short rewarming required to facilitate the trypan blue uptake is an additional stress that the imaged cells were not exposed to, so it is likely we have underestimated true viability of imaged cells.

### AntimiR uptake is concentration and time dependent

**Fig. 2:**
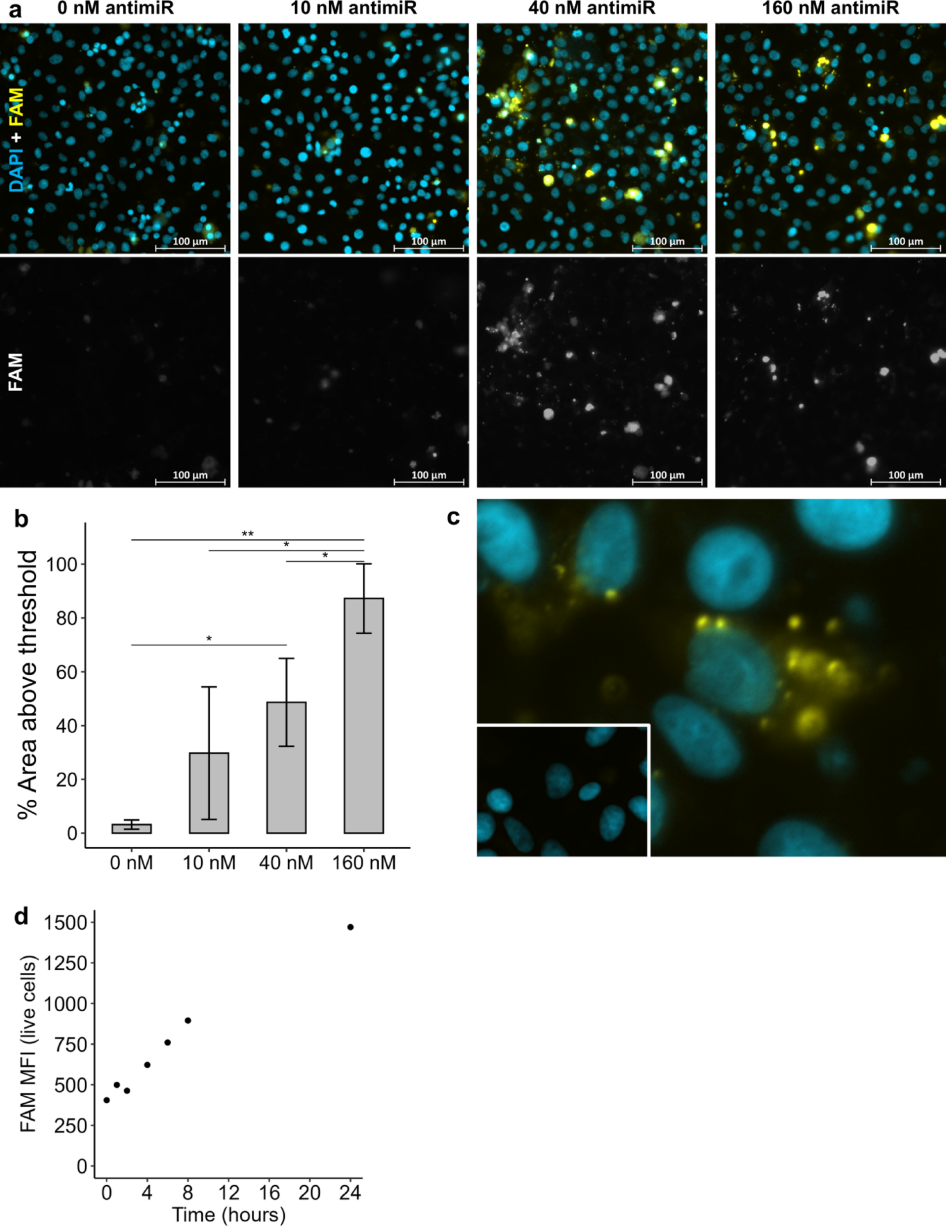
Fluorescence microscopy of naked FAM-labelled antimiR (yellow) uptake into human primary proximal epithelial cells (PTEC) after 24 h of exposure to antimiR in culture media. PTEC were maintained on transwell inserts. AntimiR was used at a concentration of 10 nM, 40 nM or 160 nM. (**a**) shows representative images (**b**) shows mean ± standard deviation area of FAM channel image that is above the threshold (set by control images) for 3 (10 nM) or 4 (40 nM, 160 nM) biological repeats. Image acquisition was standardized with the same number of images (5 for n = 1, 6 for n = 3) per condition within each biological repeat. Repeated measures ANOVA identified variance with concentration, *P* = .004. Difference between concentrations was assessed by paired t-test with Benjamini & Hochberg correction for multiple comparisons indicated with *P < .05, **P < .01. Images acquired with ZEISS AxioImager with X40 lens. A magnified area in (**c**) demonstrates vesicular pattern with 40 nM antimiR treatment (untreated in insert). (**d**) shows median fluorescent intensity (MFI) in FAM channel assessed by flow cytometry for PTEC treated for up to 24 h with 40 nM FAM-labelled antimiR (n = 1). Results displayed are for live cells (negative for amine-reactive live/dead stain). Increasing MFI indicates increasing cell uptake of antimiR.

Reports suggest that uptake of naked antimiR requires 10–100 fold higher concentrations compared to when transfection reagents are used^28^. However, use of higher concentrations may lead to toxicity^28^. To assess if lower concentrations could achieve effective uptake in PTEC, we treated cells with a range of concentrations. Twenty-four hour incubation with 10 nM FAM-labelled antimiR was not sufficient to reliably detect uptake into PTEC (Fig. 2). In contrast, both 40 nM and 160 nM treatments were sufficient to detect an increase in FAM positive areas with a mean of 49% and 87% (n = 4) of confluent areas designated positive, respectively (Fig. 2). A vesicular pattern of FAM signal was detectable, in keeping with an endocytic process (Fig. 2b,c). Flow cytometry produced a similar quantification of successful uptake for 40 nM antimiR (Supplementary Fig. 3a).

To determine speed of uptake, flow cytometry analysis of the FAM signal from PTEC (n = 1) treated with 40 nM antimiR showed FAM signal to increase steadily from 1 to 24 h of treatment (Fig. 2d). Naked delivery appears comparable to that offered by transfection reagents (Supplementary Fig. 3b–d). Data from one biological repeat suggest that naked delivery may provide a more uniform and effective delivery than transfection reagents (Supplementary Fig. 3b–d). These data demonstrate that in PTECs, unassisted uptake could be achieved with similar concentrations and speed to when transfection reagents are used, in contrast to other reports^28^.

### AntimiR colocalizes with endocytosis markers

**Fig. 3:**
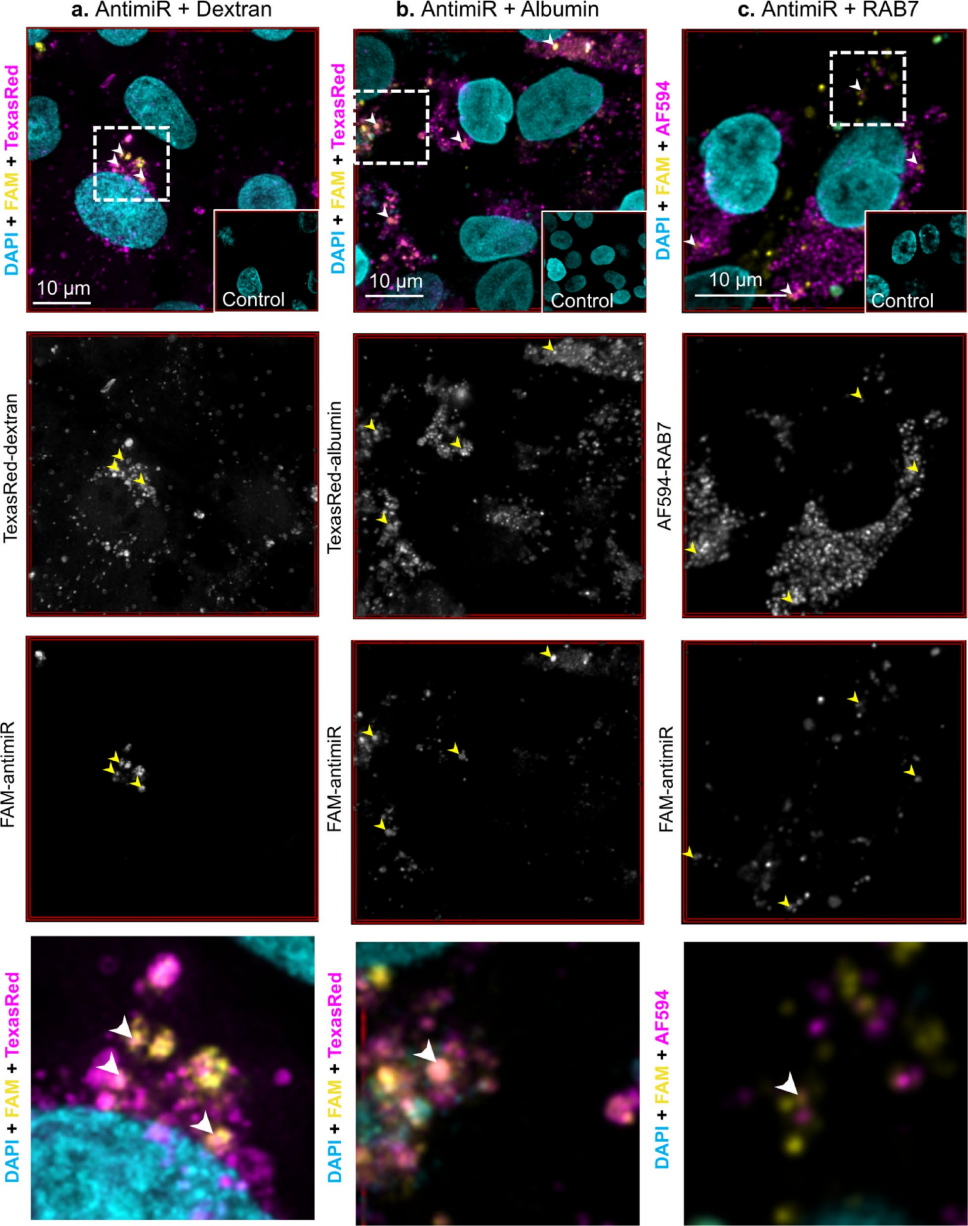
Confocal microscopy of primary human proximal tubule epithelial cells (PTEC) treated for 24 h with 40 nM FAM-labelled antimiR (yellow) and either (**a**) 1 mg/mL Texas Red-labelled dextran (magenta) or (**b**) 0.1 mg/mL Texas Red-labelled albumin (magenta). (**c**) shows PTEC treated for 8 h with 40 nM FAM-labelled antimiR with subsequent immunofluorescence for RAB7 using AF594 conjugated secondary antibody (magenta). Co-occurrence of FAM signal with marker in magenta is shown in orange. Nuclei counterstained with DAPI (cyan). Representative multichannel image shown with control in inset. All control images are of PTEC not treated with albumin, dextran or antimiR and in (**c**), an isotype control for anti-RAB7 antibody was used. Associated single channel images are shown in greyscale below. Example areas of co-occurrence indicated by white arrow heads on multichannel image and yellow arrows on single channel images, with magnified area of multi-channel image displayed on the bottom row from the dashed outlined area indicated on the full image. All images presented are max projections of deconvolved z-stacks acquired with Leica SP8-gSTED 3X using X63/1.4NA oil lens.

After a 24-h incubation, a high proportion of antimiR-associated FAM signal was found to overlap with internalized fluorescent dextran and albumin, in keeping with uptake being via an endocytic process (Table 1, Fig. 3a,b). To further explore an endocytic uptake mechanism we studied the localisation of antimiR together with the late endosome marker RAB7 and found they co-localized after 8 h of antimiR treatment (Table 1, Fig. 3c).

**Table 1:** Proportion of antimiR co-occurring with markers of endocytosis.

Marker	Mean ± SD	n	Images per repeat
Dextran	0.854 ± 0.042	3	5
Albumin	0.943 ± 0.080	2	6
RAB7	0.308 ± 0.167	3	6

Manders coefficients for proportion of fluorescent signal associated with antimiR (FAM) co-occurring with positive signal for dextran, albumin (both Texas Red labelled) or RAB7 (detected with AF594-conjugated secondary antibody). The number of biological repeats (n) and images sampled from each of these is indicated. Only pixels brighter than control images were included in co-occurrence analysis. SD, standard deviation.

### Blocking macropinocytosis reduces antimiR uptake

Macropinocytosis can be reduced by inhibiting the Na–H exchanger (NHE) on the plasma membrane^24,29^. This ion transporter is essential for the cytoplasmic alkalinisation needed for macropinosome formation as key GTPases involved (Cdc42 and Rac1) are pH sensitive^30^. EIPA is an effective inhibitor of NHE and is a recognized macropinocytosis inhibitor^24,29^. In order to determine if macropinocytosis is the major mechanism of oligonucleotide uptake, an appropriate non-toxic dose of EIPA is required as it is recognized to be cytotoxic at high concentrations^29,31^. Toxicity in response to EIPA was assessed by flow cytometry using both amine-reactive fixable live/dead stain and apoptosis assay and by fluorescence microscopy of nuclei counterstained with DAPI (Supplementary Fig. 4). Flow cytometry did not reveal any effect on viability from EIPA concentrations of 10 µM or lower (Supplementary Fig. 4). Nuclear appearance on fluorescence microscopy was found to be most sensitive at detecting toxicity with abnormal nuclear appearance demonstrated with EIPA concentrations of 2 µM and above (Supplementary Fig. 4). There was no evidence of toxicity on exposure to 500 nM EIPA (Supplementary Fig. 4). This concentration was used to assess the effect of macropinocytosis inhibition on antimiR uptake.

**Fig. 4:**
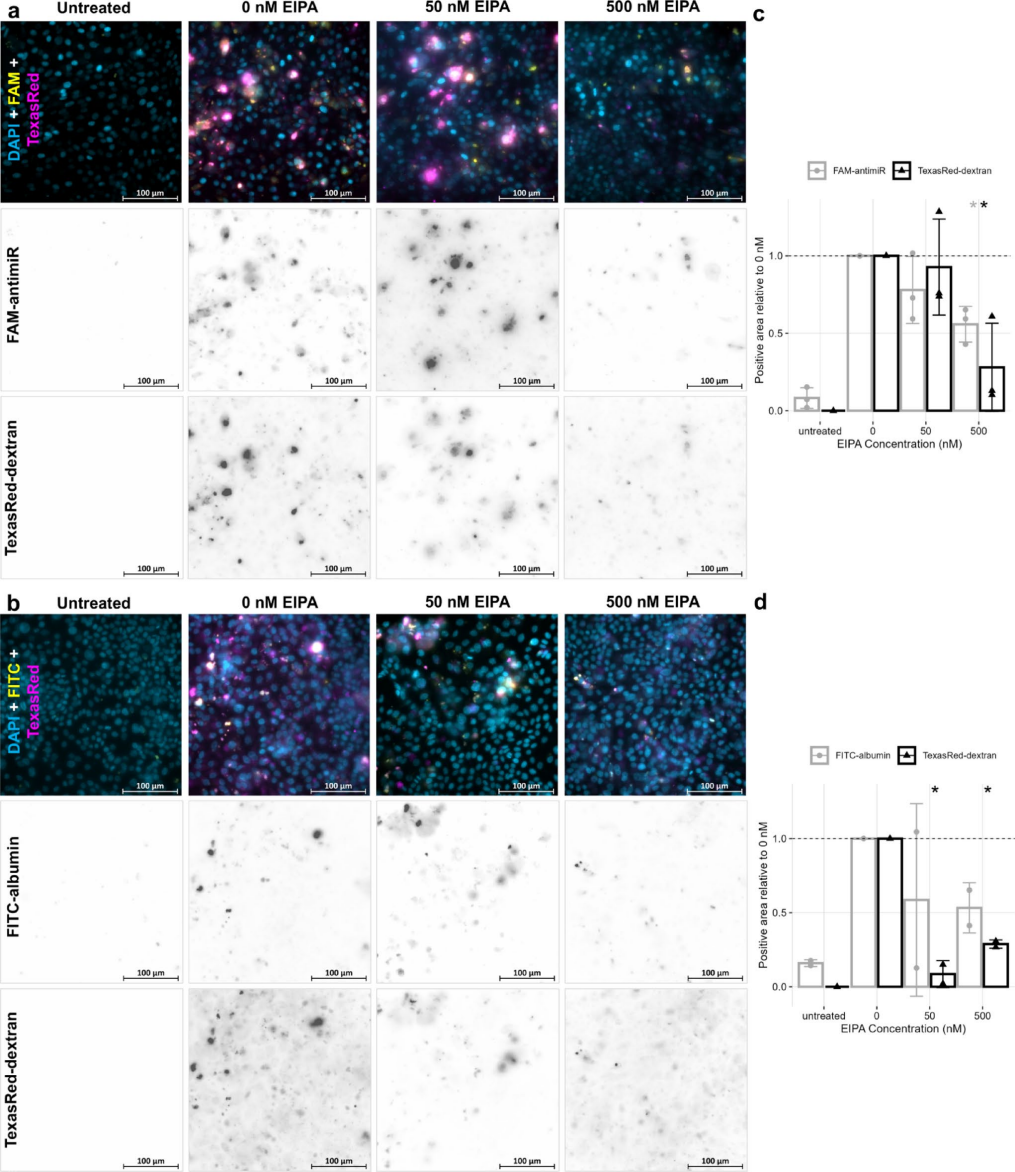
Fluorescence microscopy of human primary proximal tubule epithelial cells (PTEC) maintained on transwell inserts and treated with 1 mg/mL Texas Red-labelled dextran and either 40 nM FAM-labelled antimiR (**a**) or 0.1 mg/mL FITC-labelled albumin (**b**) for 24 h after 1 h pre-treatment with given concentration of the macropinocytosis inhibitor 5-(*N*-ethyl-*N*-isopropyl)-amiloride (EIPA) or dimethyl sulfoxide control media (0 nM EIPA). Images shown are representative of 6 images acquired at random from each condition for each biological repeat. Untreated PTEC in left column were not treated with EIPA, antimiR or dextran, as autofluorescence control. Single-channel images have been inverted for clarity. Effect of EIPA on uptake of labelled reagents used in (**a**) and (**b**) are quantified in (**c**) and (**d**) respectively as areas of FITC, FAM or Texas Red channel above threshold set by untreated controls and reported relative to 0 nM EIPA treatment. Mean ± standard deviation of (a: n = 3, b: n = 2) biological repeats are presented by bars with individual results as shapes. Statistically significant difference in EIPA treatments from 0 nM was assessed by paired t-test *P < .05. Images acquired with ZEISS AxioImager with X40 lens.

Incubation of PTEC with 500 nM EIPA reduced uptake of the endocytosis marker dextran and antimiR (Fig. 4a, c) to around 28% and 56% of control conditions, respectively. The reduction in antimiR uptake with EIPA treatment indicates macropinocytosis to be one mechanism of antimiR uptake into PTEC. There was also a trend for a reduction in albumin uptake but this was not statistically significant (Fig. 4b, d). As macropinocytosis is a non-selective process, this is expected.

### Megalin inhibition does not reduce antimiR uptake

**Fig. 5:**
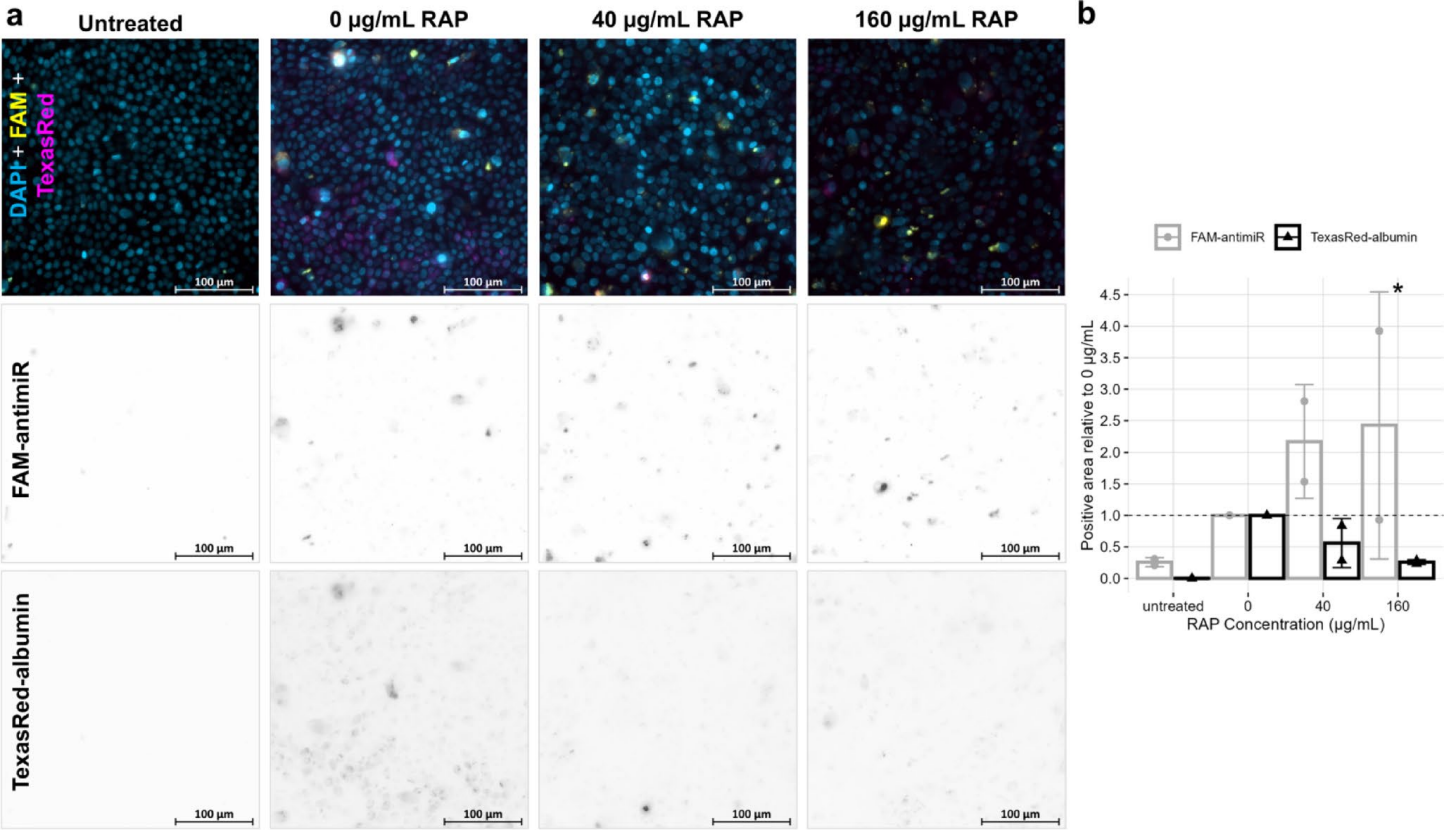
Fluorescence microscopy of human primary proximal tubule epithelial cells (PTEC) maintained on transwell inserts and treated with 160 nM FAM-labelled antimiR and 0.1 mg/mL Texas Red-labelled albumin for 24 h, after 1 h pre-treatment with given concentratin of receptor-associated protein (RAP) or control media. Images shown in (**a**) are representative of the 6 images acquired at random from each condition for each biological repeat (n = 2). Single-channel images have been inverted for clarity. Control PTEC in left column were not treated with RAP, antimiR or albumin and so show autofluorescence only in the FAM and Texas Red channels. Effect of RAP treatment on antimiR and albumin uptake are quantified in (**b**) as area of image in FAM and Texas Red channel above threshold set by untreated controls and reported relative to 0 µg/mL RAP treatment. Mean ± standard deviation of (n = 2) biological repeats are presented by bars with individual results as shapes. Difference of 40 and 160 µg/mL RAP treatments from 0 µg/mL RAP was assessed by paired t-test, *P < .05. Images acquired with ZEISS AxioImager with X40 lens.

Given the key role played by megalin (*LRP2*) in reabsorption of much of the filtrate in the proximal tubule, we next explored the effect of blocking this pathway on antimiR uptake^21^. *LRP2* siRNA knockdown did not achieve an adequate reduction in megalin protein expression to significantly impact on receptor-mediated endocytosis through this receptor (Supplementary Fig. 5), so competitive inhibition was used instead. RAP binds megalin to competitively inhibit megalin-mediated endocytosis^20,26,32^. Receptor-mediated endocytosis mediated by megalin was blocked with RAP treatment, as demonstrated by reduced albumin uptake (Fig. 5). As RAP treatment did not reduce antimiR uptake (Fig. 5), this data suggests that antimiR entry into PTEC is not by a megalin-mediated uptake mechanism. An increase in antimiR uptake was noted with RAP treatment in one repeat but given variability in results is difficult to interpret further (Fig. 5b).

## Discussion

The effects and uptake of antimiRs has largely been explored through animal models and the use of cells lines^10,15,33–36^. The study of antimiRs in proximal tubule cell lines has required the use of transfection reagents which questions the transferability of these results^33–36^. We demonstrate primary human PTECs can take up naked antimiR at concentrations as low as 40 nM, making them a useful model for studying the effects of antimiRs *in vitro*.

Our data is in keeping with previous work that the uptake mechanism is by endocytosis^9,16^. We show high levels of co-localisation with internalized albumin and dextran, both endocytic markers, and co-localisation with the late endosome marker RAB7^37^. A clear understanding of intracellular trafficking of antimiR requires more targeted assessment than what we have presented here and is important knowledge to elucidate the differences between naked and assisted delivery. Specifically, a range of earlier time points could determine association with early endosome markers, in particular. Such data, especially if coupled with functional downstream effects, has the potential to reveal important interactions between delivery method and biological activity of antimiR, that would be important to understand when choosing between techniques.

We show macropinocytosis to be involved as the inhibitor EIPA reduced antimiR uptake. Conversely, our results do not support involvement of receptor-mediated endocytosis through megalin as chemical inhibition with RAP had no effect on antimiR uptake. Interestingly, macropinocytosis has also been discussed as a possible mechanism by which therapeutics could be delivered to cells in the context of oncology^38^.Given that EIPA only partially suppressed antimiR uptake, we suspect it is not the only pathway of antimiR uptake in these cells. It would not be unique for molecules to enter PTEC by multiple endocytic pathways, as has been shown to be the case for albumin^39,40^. Takahasi et al.^41^ demonstrate that in HeLa cells the transmembrane protein SIDT2 mediates uptake of naked antimiR targeting miR-16 even at low concentrations of 20 nM. Another potential pathway is receptor-mediated endocytosis via cubilin working in partnership with amnionless^42^. More generalized disruption of clathrin-mediated endocytosis by siRNA knockdown would allow more complete assessment of the role of this branch of receptor-mediated endocytosis in antimiR uptake, not limited to that initiated by megalin. Further assessment is also needed on whether blocking one pathway increases the activity of other entry routes.

Although macropinocytosis occurs in most cells, the activity of the pathway varies greatly by cell type and condition^19,23^. Epithelial cells are recognized to be macropinocytically active and this activity can be enhanced by epithelial derived growth factor receptor stimulation^23,24^. The potential for other cell types to take up naked antimiR *in vitro* may therefore differ. We note that the concentrations we have used are closer to those used by others when involving chemical transfection reagents, as opposed to the micromolar concentrations reportedly required for gymnosis^6,7,9,43^. Our supplementary data also suggests that naked delivery may in fact results in more uniform antimiR uptake across a population of cells than that achieved by transfection reagents. This hypothesis warrants further exploration in future work and has not been fully assessed here.

It has been suggested that the uptake mechanism of oligonucleotides can affect biological activity, so it is important that *in vitro* models are as relevant as possible^44,45^. Freshly isolated human primary cells are arguably one of the more accurate *in vitro* models for studying human disease and indeed proximal tubule cell lines do not have the same transport functions as primary cells^46^. We have demonstrated that, without the use of transfection reagents, antimiR can consistently be delivered to primary human PTEC at concentrations similar to those commonly used alongside transfection reagents in cell lines^28,33^. Our future work will explore the downstream effects of a target antimiR on gene expression in primary human PTECs following naked antimiR uptake.

## Supplementary Information

Below is the link to the electronic supplementary material.

Supplementary Material 1

## Data Availability

Data supporting the findings of this study are available within the paper and its Supplementary Materials or by reasonable request from the authors.

## Abbreviations

ANOVA: Analysis of variance
antimiRs: MicroRNA inhibitors
EIPA: 5-(*N*-ethyl-*N*-isopropyl)-amiloride
FAM: Fluorescein amidites
FITC: Fluorescein isothiocyanate
miRNAs: MicroRNAs
mRNA: Messenger RNA
NHE: Na–H exchanger
PBS: Phosphate-buffered saline
PTEC: Proximal tubule epithelial cells
SD: Standard deviation
