# Supplementary material for: AntimiR uptake by human proximal tubule epithelial cells is predominantly by macropinocytosis
Source: Sci Rep. 2025 Aug 29;15:31799. doi: 10.1038/s41598-025-16522-3 (PMC12394570; doi:10.1038/s41598-025-16522-3)

AntimiR uptake by human proximal tubule epithelial cells is predominantly by macropinocytosis

Emily K Glover[1], Rolando Berlinguer-Palmini[2], Emily R Thompson,[1] Colin Wilson,[1] Laura Denby[3], Gary Reynolds[4,5], Simi Ali[1], Martin Lowe[6], Rachel Lennon[7], Neil S Sheerin[1]

[1]Translational and Clinical Research Institute, Faculty of Medical Sciences, Newcastle University, Newcastle upon Tyne, UK
[2] Bioimaging Unit, Faculty of Medical Sciences, Newcastle University, Newcastle upon Tyne, UK
[3] Centre for Cardiovascular Sciences, University of Edinburgh, UK
[4] Biosciences Institute, Newcastle University, Newcastle upon Tyne, UK
[5] Centre for Immunology and Inflammatory Diseases, Mass General Research Institute, Harvard Medical School Boston, USA
[6] School of Biological Sciences, Faculty of Biology, Medicine and Health, University of Manchester, Manchester, UK
[7] Division of Cell Matrix Biology and Regenerative Medicine, University of Manchester, Manchester, UK

Correspondence: Dr Emily K Glover (emily.glover@newcastle.ac.uk)

SUPPLEMENTARY MATERIALS

Supplementary methods

PTEC isolation and cryopreservation

Briefly, slices of cortex were minced and incubated with gentle agitation in digest mix (100 mg of reconstituted Worthington Type 2 collagenase, Lorne Labs (LS004176) per 5 g of tissue in 50 mL isolation media (RPMI (Sigma Aldrich R8758) with 5% fetal bovine serum and 2% (v/v) Penicillin/Streptomycin)), for either 2-2.5 hours at 37°C or overnight at 4°C followed by up to 2 hours at 37°C. The choice of incubation protocol was determined by logistical constraints. Digested tissue was passed through a 40 µm nylon cell strainer. The filtrate was centrifuged for 10 minutes at 1200 RPM, the supernatant was collected and centrifuged again with the pellets being combined and resuspended in isolation media. This process was repeated a further time. The combined pellets were resuspended in 25 mL of isolation media and centrifuged at 1200 RPM for 7 minutes. The loosened pellet was resuspended in isolation media and added at a volume of around 5 mL on top of two Percoll (Cytiva 17089102) gradients each composed of 1.04 density on top of 1.07 density (**Supplementary Table 1**). This was centrifuged at 3000 RPM for 25 mins and the central media layer and associated interphase beneath were extracted. The extracted layer was washed twice by centrifugation at 1200 RPM for 7 minutes.

Yield of viable cells was calculated by cell count with trypan blue exclusion test. All centrifugation was cooled at 4°C.

Cells to be cryopreserved were resuspended at 1 x 10^6 cells/mL with either 10% dimethyl sulfoxide in fetal bovine serum or with Cryo-SFM (PromoCell C-29910) and cooled to -80°C in either a Mr Frosty™ (Thermo Scientific™) or CoolCell™ (Corning®) freezing container before long-term storage in liquid nitrogen. On thawing, preservation media was removed by centrifugation at 220 RCF for 5 minutes and trypan blue viability count was repeated to inform seeding.

PTEC characterization

Three biological repeats of isolated PTEC were characterized by immunofluorescence (**Supplementary Fig.1**) to validate the isolation technique. Mouse anti-cytokeratin-19 (ThermoFisher A53-B/A2, 5 µg/mL), mouse anti-E-cadherin (BD transduction 610181, 2.5 µg/mL), rabbit anti-aquaporin-1 (Abcam ab168387, 1.95 µg/mL) and rabbit anti-tight junction protein (Sigma Aldrich HPA001636, 2µg/mL) antibodies were used in conjunction with anti-mouse Dy650 conjugated (Immunoreagents GtxMu 003 D650NHSX at 1 in 100) and anti-rabbit Dy550 conjugated (Immunoreagents GtxRb 003 D550NHSX at 1 in 100) secondary antibodies as appropriate. Rabbit (R&D rAB-105-C, 2 µg/mL) and mouse (R&D MAB0031, 5 µg/mL) isotype controls were used to demonstrate non-specific secondary binding. Immunofluorescence for cytokeratin-19 was continued for each biological repeat to confirm widespread positivity of this epithelial cell marker.

Scanning electron microcopy (SEM) was performed for one biological repeat of isolated PTEC to demonstrate microvilli and primary cilium on the apical surface (**Supplementary Fig.2**), in keeping with cells having polarized. Cultured cells were washed and fixed with 2% glutaraldehyde in Sorenson's phosphate buffer overnight at 4°C then washed twice with 0.2 M phosphate buffer. PTEC were dehydrated with increasing ethanol concentrations before proceeding to SEM.

Contamination of detached confluent monolayers by endothelial and mesenchymal cells was assessed by gating live single cells for populations of CD31 and PDGFRA positive cells on flow cytometry, respectively, using mouse anti-CD31-APC (1 µg/mL, ThermoFisher 17-0319-42), mouse anti-PDGFRα-APC (1 µg/mL, Biolegend 323511 (clone 16A1)) and mouse IgG1κ-APC isotype control (1 µg/mL, ThermoFisher 17-4714-42). Endothelial and mesenchymal cell contamination were estimated at (mean ± standard deviation, n = 10) 1.24 ± 1.15% and 0.13% ± 0.25% of cells.

Assisted antimiR delivery

Naked (unassisted) delivery of antimiR was compared with delivery assisted by Lipofectamine™ RNAiMAX transfection reagents (ThermoFisher 13778-100). Cells were treated in parallel with treatment applied to the

apical compartment for 24 hours, regardless of delivery method. As the lowest concentration to show significant naked uptake was 40 nM antimiR, this and a lower concentration of 10 nM were used to assess assisted delivery. Uptake was assessed by flow cytometry or fluorescence microscopy after 24 hours of treatment with cells seeded onto 12-well or 24-well inserts for each technique, respectively (**Supplementary Fig.3b-d**).

Transfection mix was produced by combining equal volumes of OPTI-MEM® mixed with Lipofectamine in a 50:3 ratio, with antimiR stock diluted in OPTI-MEM® to give a working concentration of 100 nM or 400 nM antimiR immediately before application. This was diluted 10-fold on application to the apical compartment with maintenance media to give final concentrations of 10 nM and 40 nM for assisted delivery. All treatments were delivered in a final apical volume of 200 µL for 24-well inserts or 500 µL for 12-well inserts.

EIPA toxicity assessment

The concentration of EIPA tolerated by PTEC without signs of toxicity was established by assessing response of PTEC to incubation with 250 nM to 100 µM EIPA for 24 hours. An amine-reactive fixable live/dead stain was used to determine viability by flow cytometry as described in main methods. Fluorescence microscopy assessed nuclear appearance, after 4% PFA fixation and counterstaining with DAPI, by both widefield fluorescence microscopy and confocal microscopy (**Supplementary Fig.4**).

Additionally, apoptosis assay (Annexin V Apoptosis Detection Kit APC eBioscience 88-8007) was performed on cells harvested with Accutase (Millipore SCR005) as per product literature and with 7-AAD (ThermoFisher Scientific 00-6993-50) used as the marker for nuclear permeability. Prepared samples were analysed within 4 hours. Single cells were gated into quadrants of necrotic (7-AAD+, APC-), late apoptotic (7-AAD+, APC+), early apoptotic (APC+, 7-AAD-) and healthy (7-AAD-, APC-) cells (**Supplementary Fig.4**).

Supplementary Table 1 Composition of Percoll gradients used for PTEC isolation

	**Stock Percoll**	**1.04 density**	**1.07 density**
**Composition**	10X HBSS 1:9 Percoll	Stock Percoll 5:12 isolation media	Stock Percoll 5:4 PBS

Stock Percoll was generated from Percoll (Cytiva 17089102) diluted with 10X HBSS (Gibco 14185045). Stock was diluted with either PBS (phosphate buffered saline) or isolation media (RPMI (Sigma Aldrich R8758) with 5% fetal bovine serum and 2% (v/v) Penicillin/Streptomycin) to generate working densities.

Supplementary figures

Supplementary Fig.1 Characterisation of isolated primary human proximal tubule epithelial cells maintained on transwell inserts by immunofluorescence for epithelial and proximal tubule markers. **a** shows aquaporin-1 (AQP1) and tight junction protein (TJP1) signal detected with dylight-550 (Dy-550; white) conjugated secondary antibody, relative to isotype control. **b** shows cytokeratin-19 and E-cadherin signal detected with dylight-650 (Dy-650; white) conjugated secondary antibody, relative to isotype control. Nuclei counterstained with DAPI (blue). Cells were PFA fixed and permeabilization with Triton-X. Widefield fluorescence microscopy images were acquired with ZEISS AxioImager with X40 lens. Representative images from one biological repeat of n=3 are displayed

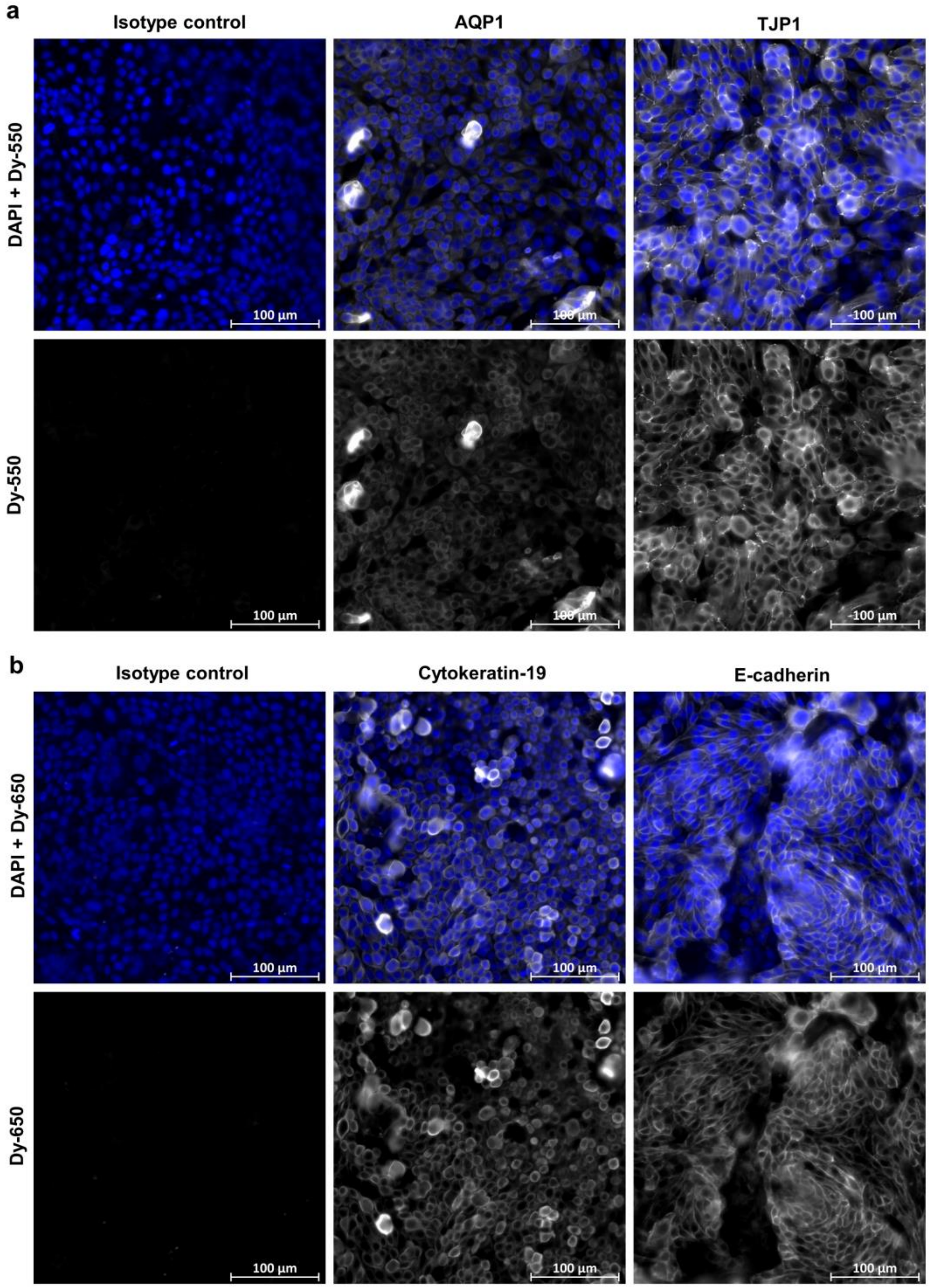

Supplementary Fig.2 Scanning electron microscopy of apical surface of isolated primary human proximal tubule epithelial cells maintained in culture on transwell inserts for 10 days. Primary cilium (large arrow) and microvilli (arrowheads) are demonstrated on the apical surface

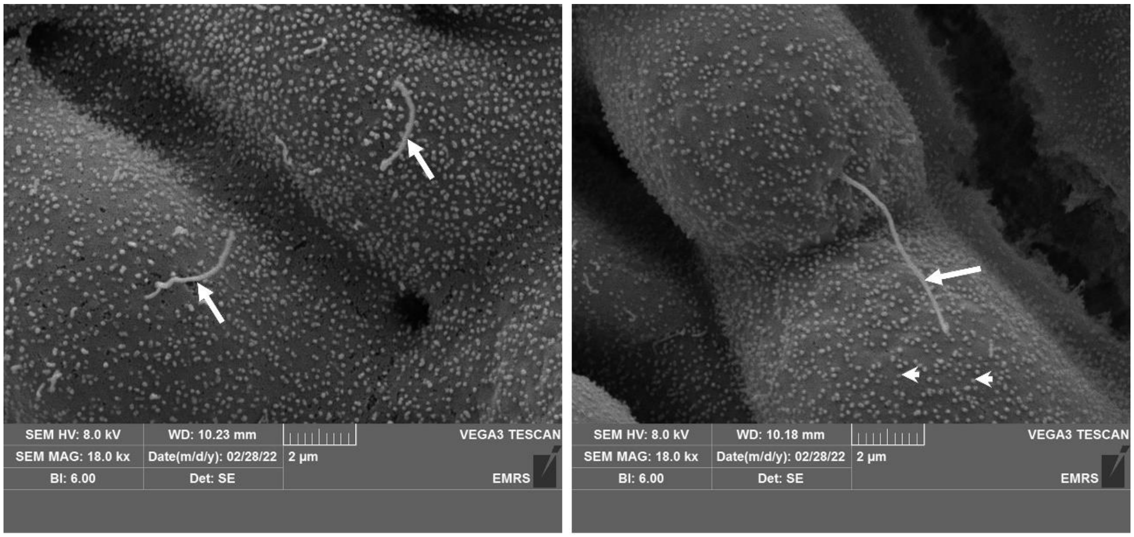

Supplementary Fig.3 Assessment of antimiR delivery to human primary proximal tubule epithelial cells (PTEC) *in vitro*. **a.** 24-hour treatment of PTEC with 40 nM FAM-labelled antimiR (black) delivered without the use of transfection reagents, resulted in increased FAM signal detected by flow cytometry. This is demonstrated by right shift of the antimiR treated cells relative to control untreated cells (grey) on the dot plot and histogram for one representative repeat. Results from biological repeats (n=3) are summarised in neighbouring graphs as mean ± standard deviation of median fluorescent intensity (MFI) for FAM or the proportion of cells within the "FAM positive" gate. Results from each biological repeat are plotted by colour for MFI to demonstrate the consistent relative positions. Difference between treatments was assessed by paired t-test, $^{**}P<.01$, with the difference by MFI approaching significance ($P=.088$). **b** shows FAM signal measured by flow cytometry after 24 hours of treatment with the given concentrations of antimiR assisted by lipofectamine delivery or antimiR delivered without transfection reagents (naked). **c** shows assessment of FAM signal (yellow) for the same conditions as in **b** but by widefield fluorescent microscopy of PFA fixed cells where nuclei are counterstained with DAPI (cyan). Images were acquired in a standardized manner using ZEISS AxioImager with X40 lens. Six images were captured at random for each condition whilst blinded to FAM signal. A representative image from each condition is shown with single channel FAM signal displayed below. **d** quantifies the data in **b** as MFI or proportion in the FAM positive gate with background signal from untreated (0 nM) cells indicated by the hashed line. The boxplot in **d** displays quantification of the 6 images acquired as per **c**, presented as the area of the image above the set threshold for FAM signal. All data in **b-d** is from the same biological repeat. Flow cytometry data was collected from fixed cells with BD Symphony A5 using the laser and filter detailed on the x axis of dot plots and histograms. All flow cytometry data displayed is for single cells gated as live by exclusion of amine-reactive dye.

a. Quantifying naked antimiR uptake by flow cytometry

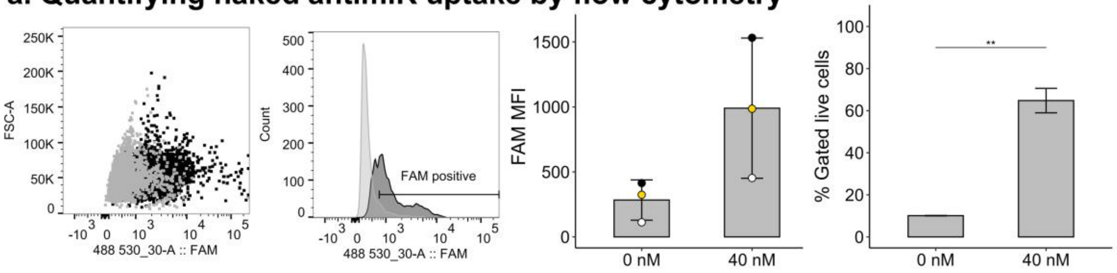

b. Effect of delivery method on antimiR uptake (flow cytometry)

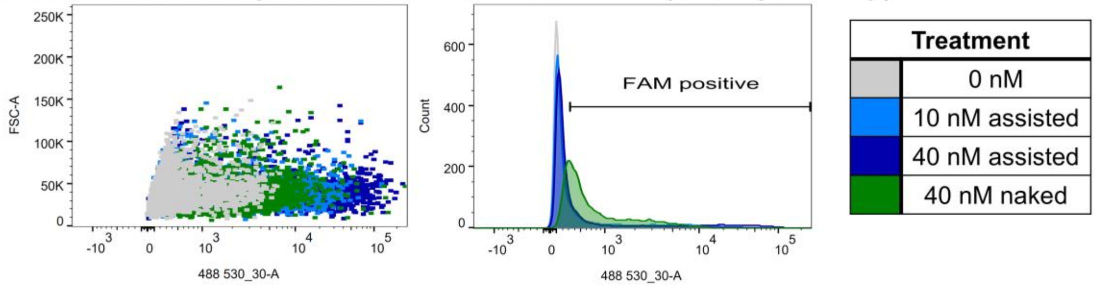

c. Effect of delivery method on antimiR uptake (fluorescence microscopy)

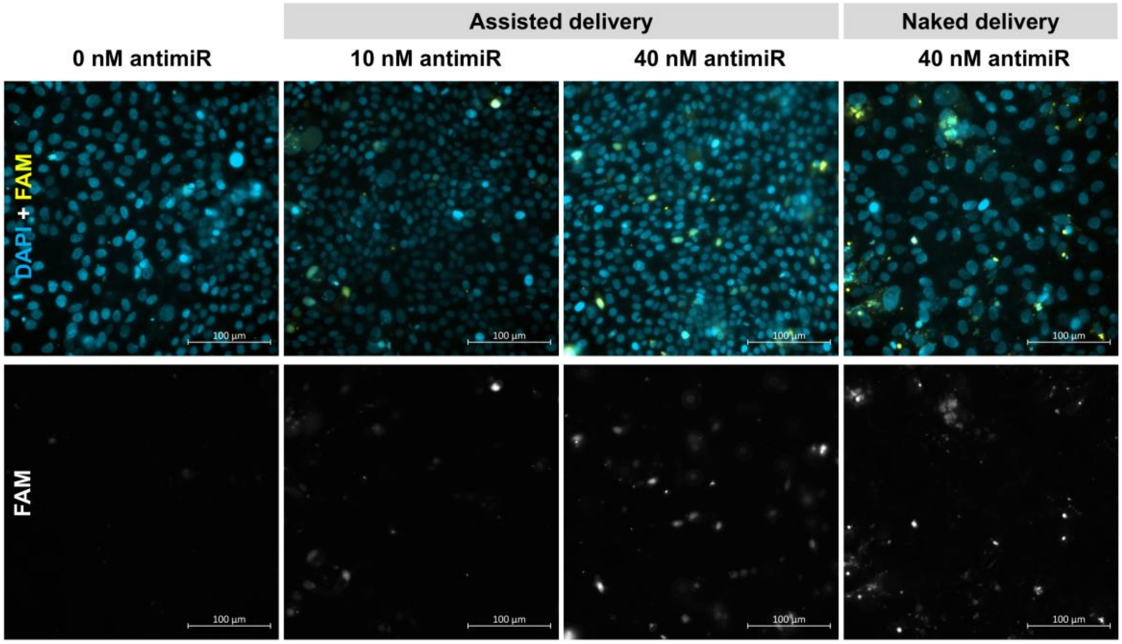

d. Quantification of antimiR uptake by delivery method

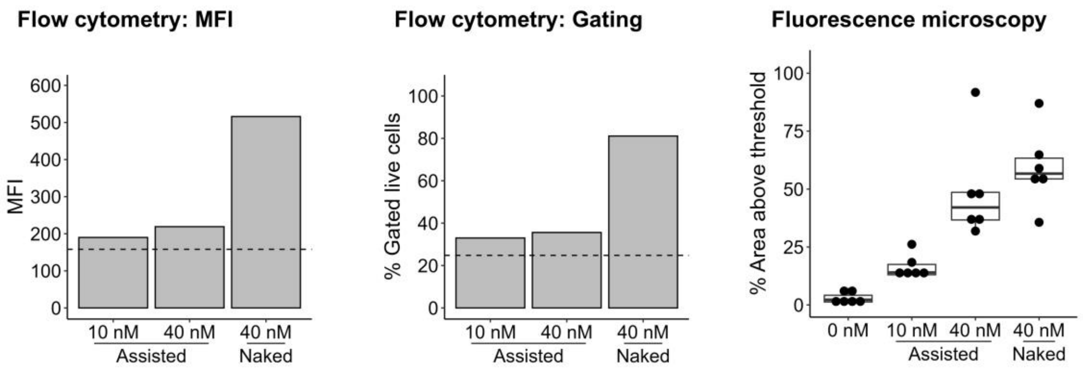

Supplementary Fig.4 Viability of human proximal tubule epithelial cells (PTEC) maintained in culture on transwell inserts and exposed to 24 hours of treatment with the macropinocytosis inhibitor 5-(N-ethyl-N-isopropyl)-amiloride (EIPA) at a range of concentrations. Viability assessed by amine-reactive live/dead stain on flow cytometry (**a**) with colour grouping results by biological repeat, found EIPA concentrations up to 10 µM were tolerated. Flow cytometry apoptosis assay results are shown in **b** (n=1) with no increase in cell death noted for EIPA concentrations down to 250 nM. Phase contrast light microscopy is shown in **c** with nuclear appearance when stained with DAPI and imaged by widefield fluorescence microscopy (WFFM) using ZEISS AxioImager with X40 lens. **d** shows nuclear appearance on fluorescence microscopy when stained with DAPI and acquired with either WFFM or confocal microscopy. Confocal images presented are max-projections of deconvolved z-stacks acquired with Leica SP8-gSTED 3X using X63/1.4NA oil lens. 4% PFA fixation was used before DAPI staining

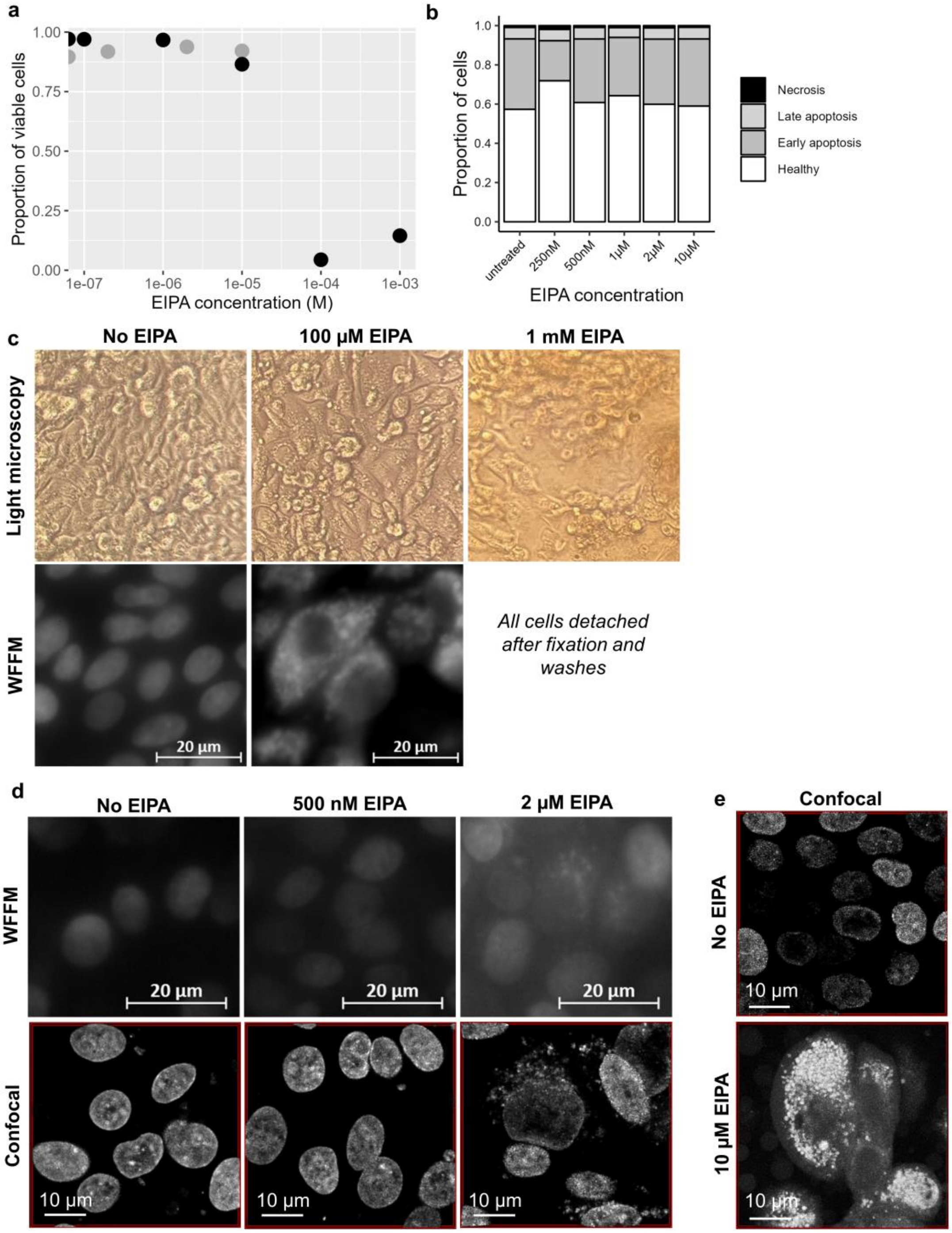

Supplementary Fig.5 Protein expression of megalin (LRP2) in human proximal tubule epithelial cells (PTEC) 120 hours after transfection with *LRP2* siRNA (light grey) is shown as median fluorescent intensity (MFI) relative to corresponding control siRNA transfected cells (dark grey) when using 200 nM siRNA in **a** and **b,** or relative to 20 0 nM control when using up to 400 nM siRNA in **c**. Paired FAM (**a**) and FITC (**b**) signal indicates uptake after a 24-hour incubation with 40 nM antimiR or 0.1 mg/ml albumin, respectively, starting 96 hours after siRNA transfection of these cells. FAM and FITC signal in LRP2 siRNA transfected cells are presented relative to control transfection. Dotted line represents values for isotype controls for anti-LRP2 antibody in megalin panels or autofluorescence in untreated cells in the case of FAM-antimiR (**a**) and FITC-albumin (**b**). Results displayed are of live single cells that have been PFA fixed and permeabilized to allow detection of total LRP2 expression. Mean ± standard deviation of n=3 biological repeats are presented in **a** with difference between groups determined by paired t-test. *P<.05. No statistics were performed in **b** or **c** as n=1

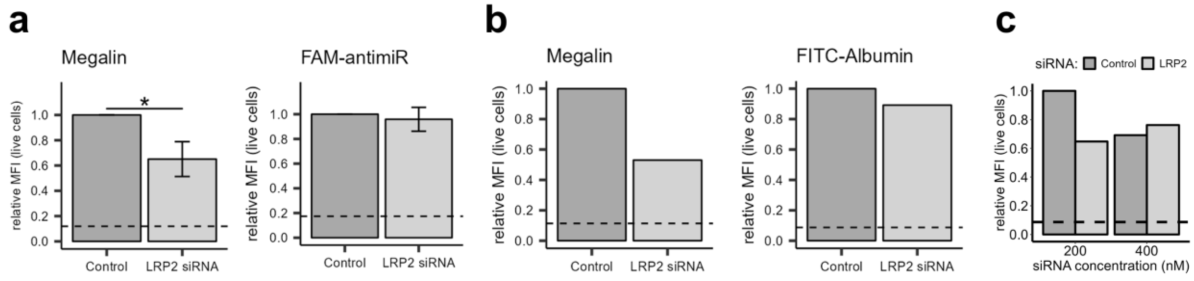

Supplement: Supplementary file 1 — Supplementary Material 1 [file 41598_2025_16522_MOESM1_ESM.pdf]
